# Chrysophanic acid shifts the differentiation tendency of BMSCs to prevent alcohol‐induced osteonecrosis of the femoral head

**DOI:** 10.1111/cpr.12871

**Published:** 2020-06-29

**Authors:** Hongping Yu, Pei Liu, Daoyu Zhu, Junhui Yin, Qianhao Yang, Yigang Huang, Yixuan Chen, Changqing Zhang, Youshui Gao

**Affiliations:** ^1^ Department of Orthopedic Surgery Shanghai Jiao Tong University Affiliated Sixth People’s Hospital Shanghai China

**Keywords:** adipogenesis, alcohol, chrysophanic acid, osteogenesis, osteonecrosis of the femoral head

## Abstract

**Objectives:**

Osteonecrosis of the femoral head (ONFH), largely caused by alcohol abuse, is a refractory bone disease characterized by the impaired capacity of osteogenic differentiation of bone mesenchymal stem cells (BMSCs), as well as the disordered adipocyte accumulation. Chrysophanic acid (CPA) is a natural anthraquinone which has lipid regulation and bone protection capacity. The aim of this study was to reveal the potential function of CPA and the underlying mechanisms for the alcohol‐induced ONFH.

**Materials and Methods:**

The effects of alcohol and CPA on BMSCs were investigated by cell proliferation, induced differentiation assays and immunofluorescent staining. Meanwhile, the function of PI3K/AKT and AMPK pathway was investigated in the process of osteogenic and adipogenic differentiation, respectively. Furthermore, we established the rat model of alcohol‐induced ONFH to reveal the pharmacotherapeutic effect of CPA in vivo using radiographical and histopathological methods.

**Results:**

In vitro, alcohol significantly inhibited the proliferation and osteogenic differentiation of BMSCs but stimulated the adipogenic differentiation. However, CPA could counteract the anti‐osteogenesis of alcohol partly *via* PI3K/AKT pathway and retard the promotion of alcohol‐induced adipogenesis *via* AMPK pathway. In vivo, radiographical and histopathological findings showed that CPA could alleviate alcohol‐induced ONFH and substantially restore the bone volume.

**Conclusions:**

We demonstrated that CPA ameliorated alcohol‐induced ONFH possibly *via* regulating the differentiation tendency of BMSCs. Hence, CPA may become a beneficial herb extract to alleviate alcohol‐induced ONFH.

## Graphical abstract

Alcohol impairs the vitality of bone homeostasis within the femoral head, which usually undergoes osteonecrosis with substantial adverse influence on the quality of life. Fortunately, chrysophanic acid (CPA), a natural anthraquinone, can shift the differentiation tendency of bone mesenchymal stem cells, thus to strengthen the capacity of bone formation and retard the pathology of adipocyte accumulation. CPA might become a beneficial herb extract to alleviate alcohol‐induced bone diseases.

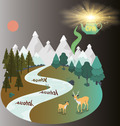

## 1 INTRODUCTION

ONFH is a refractory bone disease characterized by the death of bone cells and the sparsity of osseous microstructure, which largely progresses to the biomechanical failure with a pathological cartilage delamination and deformation of the hip joint.^1^, ^2^ Up to now, the exact mechanism of the non‐traumatic ONFH has not been fully demonstrated. Nevertheless, several risk factors of the non‐traumatic ONFH have been revealed, including corticosteroids medication, alcohol (AL) abuse, lipid metabolism disorders and autoimmune diseases.^3^ Although alcohol drinking is one of the most common aetiologies for ONFH, especially in the eastern Asia, the exact pathway of alcohol‐induced interruption on the bone homoeostasis of the femoral head needs further elucidation.^4^, ^5^

BMSCs have high proliferative potential and the ability to differentiate into osteoblasts, chondrocytes and adipocytes.^6^ BMSCs are considered as the precursor cells of osteoblasts, thus critically influencing bone growth, regeneration and remodelling.^7^ BMSCs pools are impaired, and the osteoblasts show significant abnormalities in patients with ONFH.^8^ Therefore, some researchers hypothesized that ONFH might be a disease of BMSCs. Intriguingly, accumulating reports indicated that alcohol impairs bone homoeostasis *via* a direct inhibition on BMSCs. Particularly, alcohol significantly inhibits proliferation and DNA synthesis of the osteoprogenitor cells.^9^ Moreover, alcohol critically retards the formation of mineralization nodes and the osteogenic differentiation of BMSCs.^10^ On the contrary, alcohol tends to induce BMSCs into adipocytes.^11^ Intriguingly, intramedullary fat accumulation and trabecular sparsity are essential pathological features of ONFH.^12^, ^13^ Therefore, the disturbed differentiation tendency of BMSCs into osteoblasts and adipocytes might substantially contribute to the aetiological foundation of the alcohol‐induced ONFH.

Recently, an increasing interest has risen for the treatment of intractable bone diseases with traditional Chinese medicine (TCM).^14^ Our previous studies have indicated that muscone, cordycepin and osthole have beneficial effects for ONFH.^15^, ^16^, ^17^ CPA, also known as chrysophanol and 1,8‐dihydroxy‐3‐methyl‐anthraquinone, can be extracted from various herbs, including *Rumex crispus, Polygonum multiflorum* and *Cassia obtusifolia*.^18^, ^19^ CPA has been revealed to possess many beneficial pharmacological effects, such as lipid regulation, neuroprotection, anti‐cancer, anti‐inflammation, hepatoprotection and anti‐obesity function.^19^, ^20^, ^21^, ^22^, ^23^ It is worth noting that the ethanol extract of *Rumex crispus* root increases alkaline phosphatase (ALP) and bone nodule formation in MG‐63 cells,^24^ and the water extract of *Rumex crispus* root inhibits osteoclast differentiation *via* RANKL pathway and promotes osteoblast mineralization *via* extracellular signal‐regulated kinase (ERK) phosphorylation.^24^ Moreover, CPA remarkably inhibits adipogenesis *via* AMPK pathway both in vivo and in vitro.^25^ Therefore, we hypothesized that CPA might exert a potential protection for the alcohol‐induced ONFH *via* regulating the differentiation tendency of BMSCs. In this study, for the first time, we demonstrated that CPA could modulate the differentiation tendency of BMSCs, alleviate the morphological features of alcohol‐induced ONFH and substantially restore the bone volume within the femoral head.

## 2 MATERIALS AND METHODS

### 2.1 Cell culture

We extracted BMSCs from tibias and femurs of 4‐week‐old Sprague‐Dawley rats based on the previous protocol.^17^, ^26^ BMSCs were incubated in α‐minimum essential medium (α‐MEM) (Gibco) with 10% foetal bovine serum (FBS) (Gibco), 1% penicillin/streptomycin (Invitrogen) at 37°C with 5% CO_2_ in a humidified atmosphere. The cells used in the whole study were between the 3^rd^ to the 7^th^ passage.

### 2.2 Cell proliferation detection

The doses of AL (50 mM) (Sinopharm) and CPA (1, 5, 10 μM) (Selleck) were according to the previous investigations.^8^, ^20^ In brief, BMSCs were seeded in 96‐well plates at 5 × 10^3^ per well in 100 μL medium with additional AL or CPA. To detect BMSCs proliferation, 90 μL medium and 10 μL CCK‐8 solution (Beyotime) were added to each well and then cultured in 37 ℃.

### 2.3 Osteogenesis assay

1 × 10^5^ of BMSCs were seeded in 24‐well plates. When cells came to 80% confluence, osteogenic medium (Cyagen) was used to induce osteogenic differentiation and refreshed every 2 days. Alizarin red staining (ARS) was conducted and quantified at Day 21. ALP staining and its activity detection were performed at Day 7 and Day 14. All images were captured by a LEICA microscope, and the ALP activity was detected at 560 nm by a multifunctional microplate reader.

### 2.4 Adipogenesis assay

2 × 10^5^ cells were seeded in 6‐well plates. When cells reached 80% confluence, adipogenic medium (Cyagen) was used to induce adipogenic differentiation and refreshed according to the manufacturer's protocol. Oil Red O staining was conducted and quantified at Day 21.

### 2.5 Quantitative real‐time polymerase chain reaction assay (QPCR)

Bone mesenchymal stem cells (BMSCs) were incubated in respective medium for 48 h, and then, total RNA was extracted based on the manufacturer's protocol (EZBioscience). Reverse transcriptase reactions included the purified total RNA and 50 nM RT primer, with M‐MLV reverse transcriptase (EZBioscience) used. QPCR was performed by an ABI 7900T system. GAPDH was set as the reference gene of RNA expression. The primers are listed in Table 1.

**Table 1:** The RT‐PCR primers used in this study

Gene	Forward primers	Reverse primers
COL I	CTCAAGAAGTCCCTGCTCCTC	GACTGTCTTGCCCCAAGTTC
OCN	GCATCCTTGGCTTTGCAGTC	AGTGTTTGCTGTAATGCGCC
OPN	CCGTTTAGGGCATGTGTTGC	CCGTCCATACTTTCGAGGCA
PPARγ	CCGCATTTTTCAAGGGTGCC	CCGCAGGCTTTTGAGGAACT
LPL	CTGAGTGCTGGGAGCTTGAT	GACGTGTGACAGAGGTACGG
Leptin	CTGAGTGCTGGGAGCTTGAT	GACGTGTGACAGAGGTACGG
GAPDH	CAGGTTGTCTCCTGCGACTT	TATGGGGGTCTGGGATGGAA

### 2.6 Western blotting

In brief, BMSCs were lysed by cell RIPA lysis buffer (Beyotime) with 1 mM phenylmethylsulfonyl fluoride (PMSF). Equal proteins (25 μg) were electrophoresed on 10% SDS‐PAGE, transferred to polyvinylidene fluoride (PVDF) membranes and blocked with skim milk, and incubated with the primary antibodies against osteocalcin (OCN), Runx2, GAPDH, PI3K, AKT, p‐AKT, peroxisome proliferators‐activated receptor‐γ (PPARγ), AMPK and p‐AMPK (CST) in tris‐buffered saline Tween‐20 (TBST). Then, the membranes were washed and incubated with an HRP‐conjugated second antibody. Finally, the PVDF membranes were washed and reacted with the ECL kit (Thermo Scientific). Scanning densitometry was used to quantify the signals.

### 2.7 Immunofluorescent staining

Bone mesenchymal stem cells (BMSCs) were seeded in confocal dishes for 24 h with α‐MEM. Subsequently, cells were cultured with respective medium containing AL (50 mM) or additional CPA (10 μM) for 48 h. Then, cells were washed and fixed, permeabilized, and incubated with primary antibodies against collagen I (COL I) (CST) and OCN (CST). BMSCs were washed and incubated with the Alexa Fluor^TM^ 488 secondary antibodies. Finally, DAPI and phalloidine were used to stain BMSCs nucleus and cytoskeleton, respectively.

### 2.8 In vivo studies

Thirty 8‐week‐old male Sprague‐Dawley rats were used to establish the alcohol‐induced ONFH model with the approval from the Animal Research Committee at Shanghai Sixth People's Hospital. The rats were randomly divided into three groups (n = 10): (1) control group, (2) AL group and (3) AL + CPA group. All animals were allowed to adapt to the Lieber‐DeCarli liquid diet for one week. For the next 6 weeks, the AL group had an ethanol‐containing Lieber‐DeCarli liquid diet,^27^, ^28^ while animals in the AL + CPA group had the same ethanol diet with supplementary CPA (5 mg·kg^‐1^·day^‐1^) by intraperitoneal injection,^19^ and animals in the control group were fed with the normal Lieber‐DeCarli liquid diet without ethanol. All rats had free access to the diets, which were refreshed daily. Intraperitoneal injections of 20 mg·kg^‐1^ tetracycline (Aladdin), 10 mg·kg^‐1^ calcein‐AM (Aladdin) and 30 mg·kg^‐1^ alizarin red S (Aladdin) were performed at weeks 0, 2 and 4 during the experiment for fluorescence staining.^29^

### 2.9 Micro‐CT scanning

All rats were sacrificed under the general anaesthesia, and bilateral femoral heads were harvested and fixed in 4% paraformaldehyde. All femoral heads were scanned by a micro‐CT scanner (SkyScan 1176). The CTVol software was used to manage and reconstruct the images. Bone parameters were analysed by the reconstructed images, including trabecular number (Tb.N), trabecular bone volume fraction (BV/TV), bone mineral density (BMD) and trabecular thickness (Tb.Th).

### 2.10 Histological and immunohistochemical staining

Femoral heads were decalcified, embedded in paraffin, sectioned into 5 μm thick slices and stained with haematoxylin and eosin (H&E). For immunohistochemical staining, slides were deparaffinized, antigen retrieved, blocked and incubated with the primary antibodies of COL I and OCN, and the relevant biotinylated secondary antibodies. Finally, slides were stained with DAB and further counterstained with haematoxylin.

### 2.11 Apoptosis assay

Cellular apoptosis within the femoral head was detected by TdT‐mediated dUTP nick‐end labelling (TUNEL) technique. When the section was deparaffinized and antigen retrieved, the TUNEL staining was undertaken based on the manufacture's protocols (Beyotime).

### 2.12 Statistical analysis

All data were shown as means ± standard error of the mean (SEM). Statistical differences were determined by the Bonferroni's post hoc test (one‐way ANOVA) or Student's t test in SPSS 18 (IBM). ^*^P < .05, ^**^P < .01, and ^***^P < .001 were considered to have statistical significance.

## 3 RESULTS

### 3.1 CPA abolished the inhibition of alcohol on osteogenic differentiation of BMSCs

**Figure 1:**
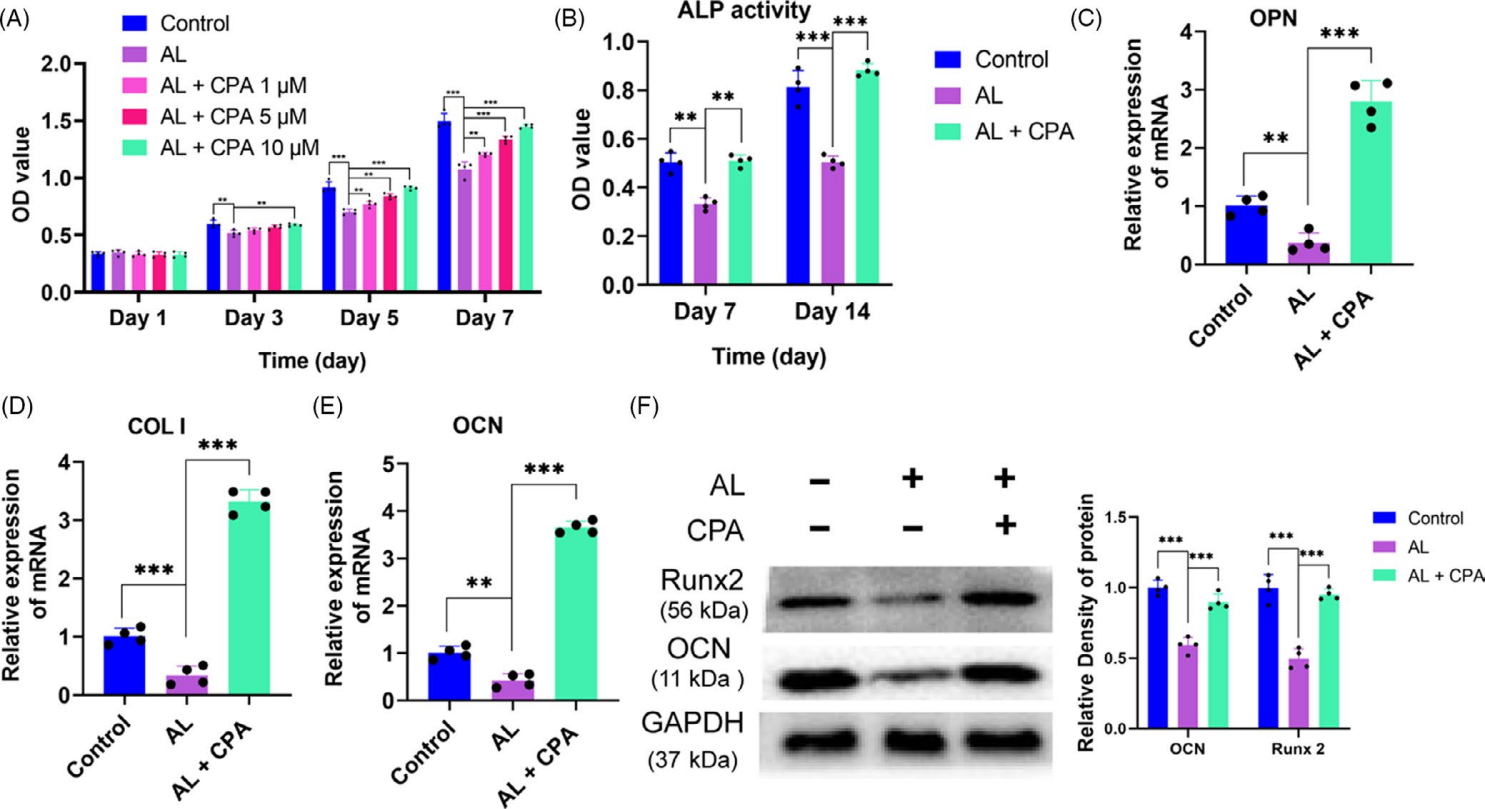
Alcohol‐induced inhibition on cell proliferation and osteogenic differentiation of BMSCs was alleviated by CPA. (A) The proliferation capacity of BMSCs treated with alcohol and CPA was evaluated by CCK‐8 assay. (B) The ALP activity of BMSCs treated with alcohol and CPA was detected at Day 7 and Day 14. (C‐E) COL I, OCN and OPN mRNA of BMSCs treated with alcohol and CPA. (F) Both Runx2 and OCN were decreased by alcohol but substantially strengthened by CPA in BMSCs

CCK‐8 assay revealed that BMSCs proliferation was significantly inhibited by 50 mM alcohol at Day 3, Day 5 and Day 7. CPA, by contrast, could substantially counteract the inhibition of alcohol on BMSCs proliferation in a dose‐dependent manner at Day 5 and Day 7. Notably, 10 μΜ CPA could abolish the inhibition of alcohol as early as on Day 3 and yielded the best rescue effect on Day 5 and Day 7. Therefore, we used 10 μΜ CPA in the following experiment (Figure 1A).

**Figure 2:**
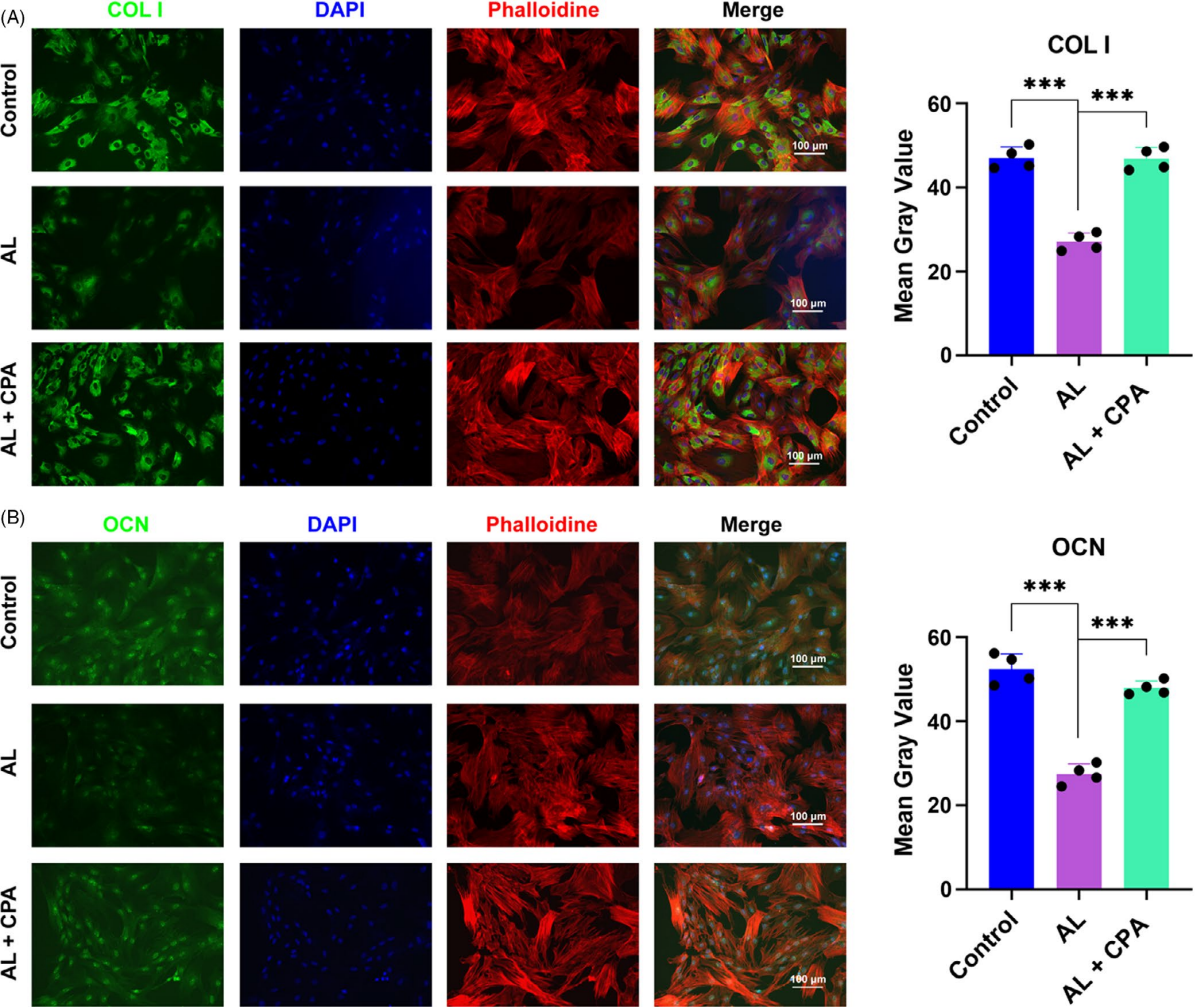
Immunofluorescent staining to show the effect of alcohol and CPA in osteogenic differentiation of BMSCs. Immunofluorescence of COL I (A) and OCN (B) showed CPA significantly restored the osteogenesis capacity of BMSCs, which was critically suppressed by alcohol. BMSCs were treated with alcohol and CPA for 48 h. Cytoskeletons and the nucleus were stained with phalloidine (red) and DAPI (blue)

**Figure 3:**
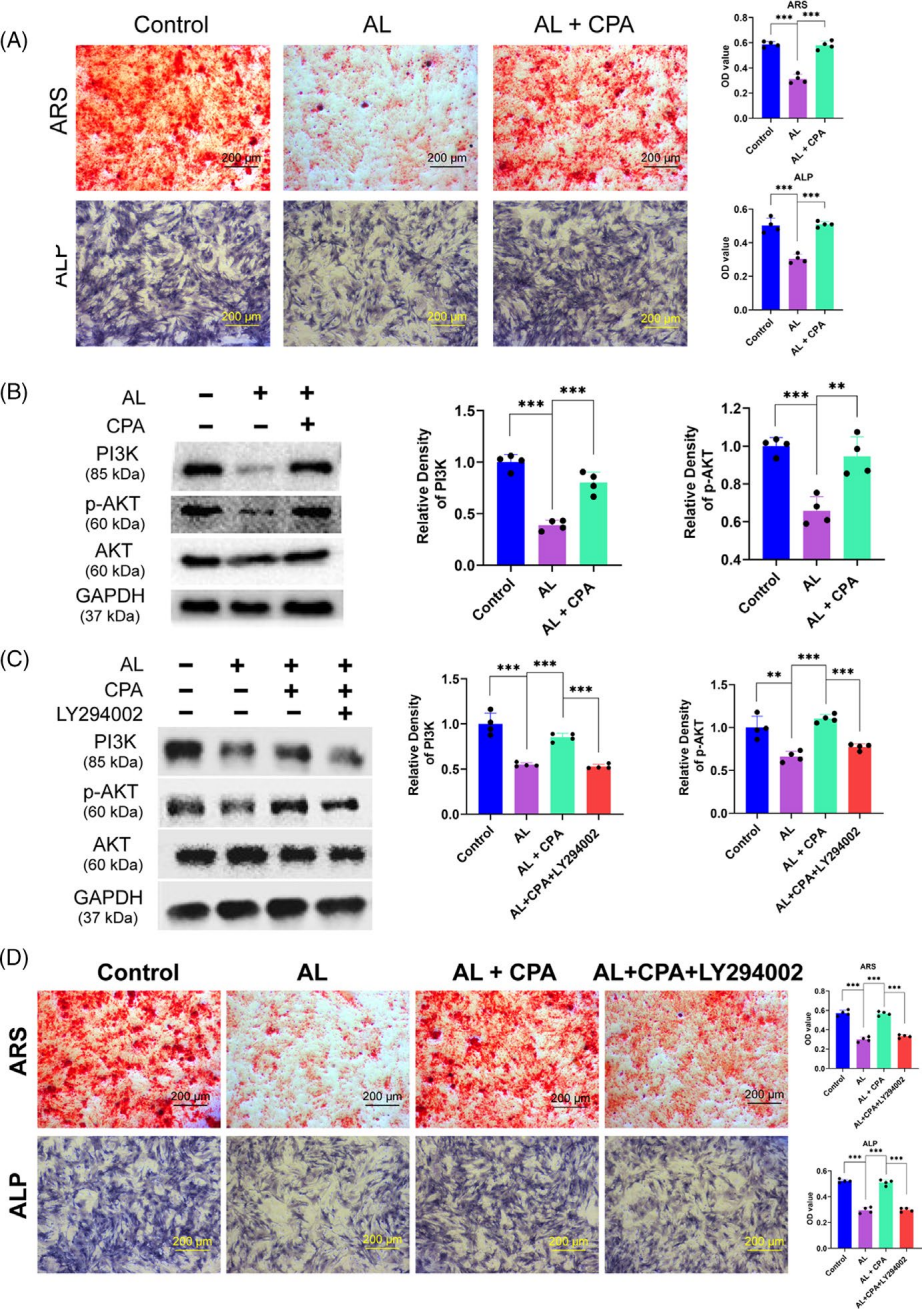
The beneficial effects of CPA in rescuing osteogenic differentiation of BMSCs were attributed to the PI3K/AKT pathway in part. (A) ARS and ALP staining of BMSCs incubated with alcohol and CPA for 21 days and 7 days, respectively. (B) PI3K and p‐AKT were dramatically decreased by alcohol, but both were rescued by CPA in BMSCs for 48 h. (C) Selective PI3K/AKT antagonist LY294002 abolished the protective effects of CPA. (D) The rescue effect of CPA on impaired osteogenesis of BMSCs was largely abolished by LY294002, as evidenced by ARS and ALP staining

Subsequently, the osteogenesis assay indicated that alcohol could remarkably inhibit the ALP activity at Day 7 and Day 14, while co‐treatment with CPA could substantially abolish the inhibition of alcohol (Figure 1B). As the osteogenic‐associated markers, the gene expression of COL I, OCN and OPN of BMSCs was distinctly decreased by alcohol, while CPA could reverse the inhibition and promote genes expression to a much higher level (Figure 1C‐1E). Moreover, the protein expression of OCN and Runx2 were remarkably decreased by alcohol; however, the inhibitory effect was distinctly rescued by supplementary CPA (Figure 1F). Immunofluorescence staining demonstrated that the intensity of COL I and OCN was apparently decreased by alcohol; however, CPA could reverse the alcohol‐induced inhibition on COL I and OCN expression of BMSCs (Figure 2A and 2B). Finally, the ARS and ALP staining indicated that both mineralization nodes and ALP activity were significantly decreased by alcohol, while CPA treatment could critically restore the capacity of osteogenesis when BMSCs were cocultured in 50 mM ethanol. (Figure 3A).

### 3.2 CPA rescued alcohol‐induced anti‐osteogenesis *via* PI3K/AKT pathway

PI3K/AKT signalling pathway is vital to cell development and differentiation. The results demonstrated that 50 mM alcohol dramatically decreased the level of PI3K and phosphorylated AKT. However, co‐treatment with CPA could restore the expression of PI3K and p‐AKT within BMSCs against alcohol (Figure 3B). LY294002, a selective inhibitor, was used to block PI3K/AKT pathway to detect its role in the osteoprotective effect of CPA. It was revealed that 500 nM LY294002 distinctly decreased the upregulation of PI3K and p‐AKT when alcohol‐treated BMSCs were further cultured with CPA (Figure 3C). Next, ARS and ALP staining were conducted to observe the role of PI3K/AKT signalling in morphological performance. The results showed that LY294002 dramatically abrogated the preventive effect of CPA on the alcohol‐induced loss of extracellular mineralization (Figure 3D).

### 3.3 CPA counteracted the alcohol‐induced adipogenesis of BMSCs

**Figure 4:**
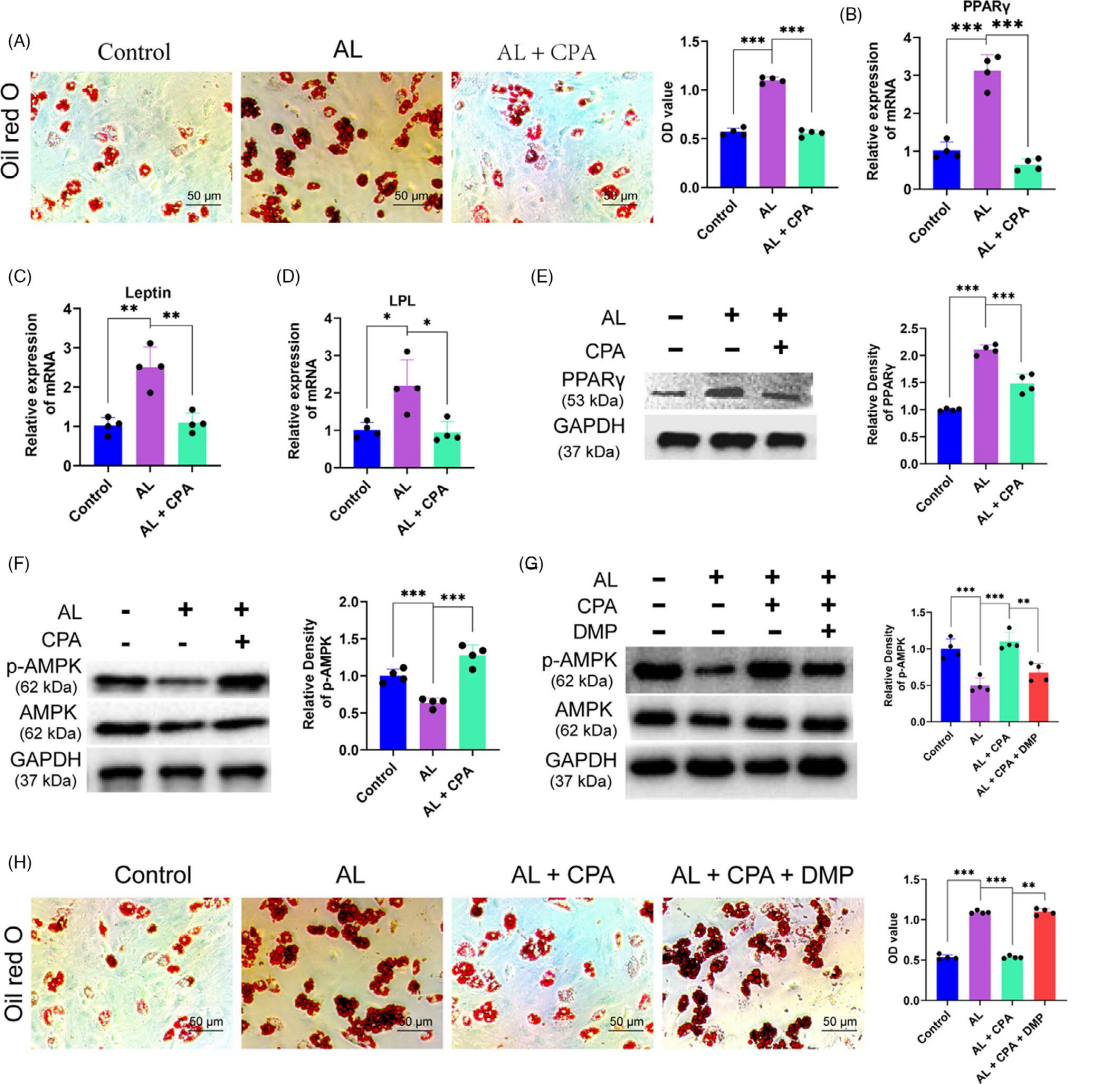
Rising adipogenic differentiation of BMSCs by alcohol was retarded by CPA administration. (A) Oil red O staining of BMSCs treated with alcohol and CPA for 21 days. (B‐D) PPARγ, leptin and LPL were upregulated in BMSCs after 7‐day incubation with alcohol; however, the promotion of adipogenesis biomarkers was critically abolished by CPA. (E) PPARγ was upregulated by alcohol and retarded by CPA in BMSCs. (F) The level of p‐AMPK was significantly inhibited by alcohol but greatly recovered by CPA treatment. (G) Selective AMPK antagonist DMP weakened the protective effects of CPA. (H) The rescue effect of CPA functions *via* AMPK pathway to retard adipogenic differentiation of BMSCs stimulated by alcohol

Adipogenesis assay indicated that alcohol remarkably promoted the adipogenesis of BMSCs with a myriad of lipid vacuoles formation; however, CPA could significantly retard the promotion of adipogenic differentiation of BMSCs by alcohol (Figure 4A). The levels of adipokines of PPARγ, lipoprotein lipase (LPL) and leptin in BMSCs treated with alcohol and CPA were detected by QPCR. As shown in Figure 4B‐4D, the gene expression levels of PPARγ, leptin and LPL were significantly upregulated by alcohol, while CPA dramatically counteracts the upregulation. Moreover, alcohol substantially increased the protein level of PPARγ in BMSCs, whereas the stimulation was distinctly abolished by CPA co‐treatment (Figure 4E).

### 3.4 CPA retarded the alcohol‐irritated adipogenesis *via* AMPK pathway

AMPK signalling pathway has a key role in the fatty acid metabolism. Western blot demonstrated that alcohol remarkably decreased the level of p‐AMPK (Figure 4F). However, co‐treatment with CPA could reverse the inhibition of alcohol on p‐AMPK in BMSCs (Figure 4F). DMP, a selective antagonist, was used to investigate the role of AMPK signalling in the adipogenesis of BMSCs cocultured with alcohol and CPA. The result demonstrated that 0.1 μM DMP distinctly decreased the upregulation of p‐AMPK when BMSCs were treated with alcohol and CPA (Figure 4G). Finally, Oil Red O staining was performed to further investigate the exact role of AMPK signalling. The result indicated that DMP dramatically abolished the preventive effect of CPA on the alcohol‐induced extracellular adipogenesis in BMSCs (Figure 4H).

### 3.5 CPA alleviated alcohol‐induced ONFH in the rat model

**Figure 5:**
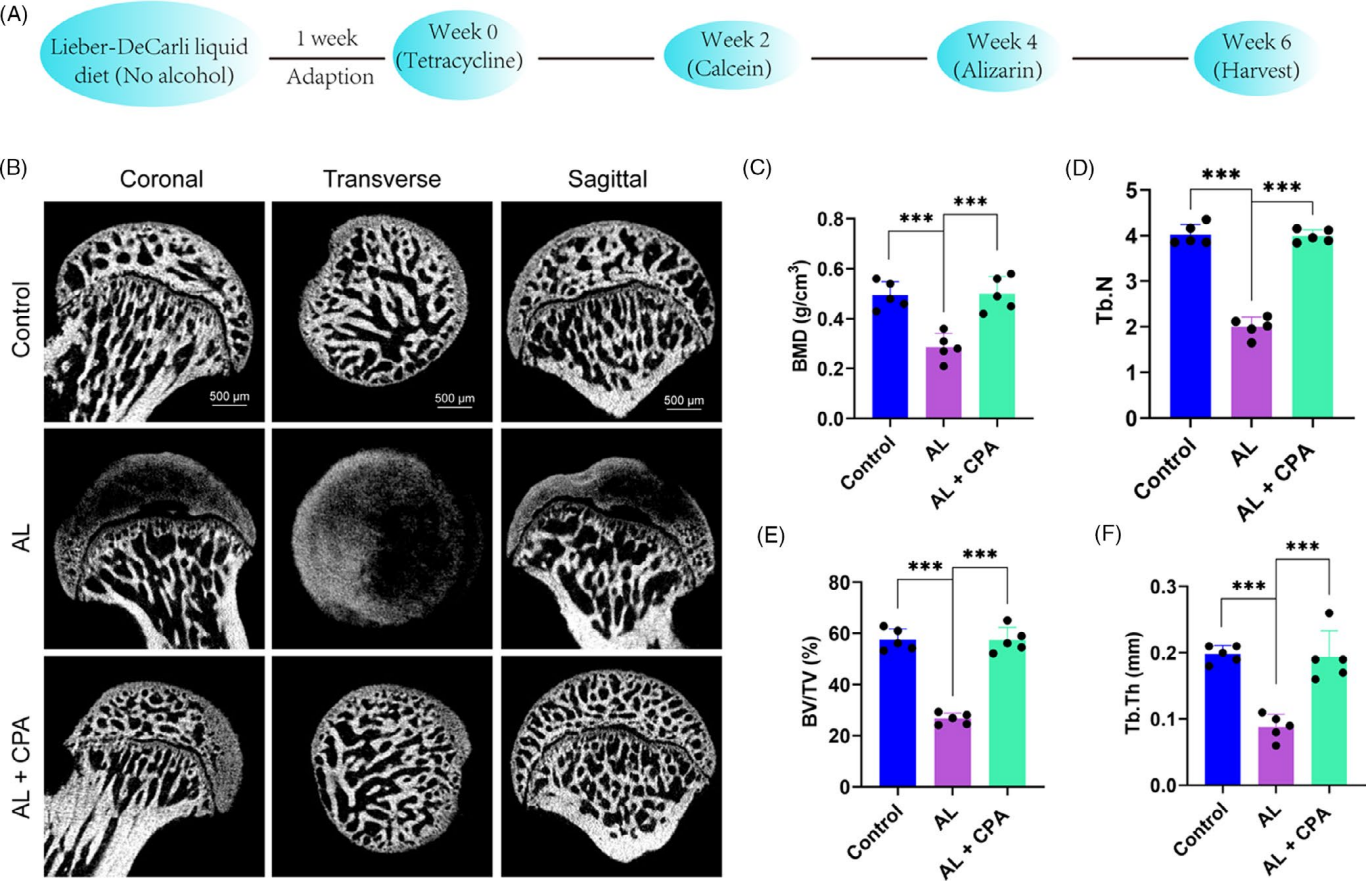
Micro‐CT scanning and analysis of the femoral head. (A) The schematic diagram of the in vivo experimental procedure. All animals were allowed to alcohol‐free Lieber‐DeCarli diet for 1 week. After that, the rats were divided with respective treatment protocol. Tetracycline, calcein and alizarin red S were used to monitor dynamic bone formation. (B) Micro‐CT scanning images of the femoral head from animals of different groups. The rats in the AL group had dramatically impaired subchondral trabecular bone, which was critically restored by the pharmacotherapy of CPA. (C‐F) BMD, Tb.N, BV/TV and Tb.Th were calculated based on the reconstructed images

The experimental procedure was illustrated in Figure 5A. Following the specimen harvest at week 6, the osseous structure of the femoral head was evaluated by micro‐CT scanning. In brief, 8 of 10 rats in the alcohol group had obvious signs of osteonecrosis, while only two rats had mild signs of osteonecrosis in the alcohol + CPA group. Notably, no signs of osteonecrosis were observed in the control group (Figure 5B). The detrimental effect of alcohol and the beneficial role of CPA were further studied by imaging analysis. These vital bone parameters, BMD, Tb.N, BV/TV and Tb.Th, in the alcohol group were significantly decreased compared to the control group (Figure 5C‐5F). However, co‐treatment with CPA obviously upregulated these bone parameters of the femoral head.

**Figure 6:**
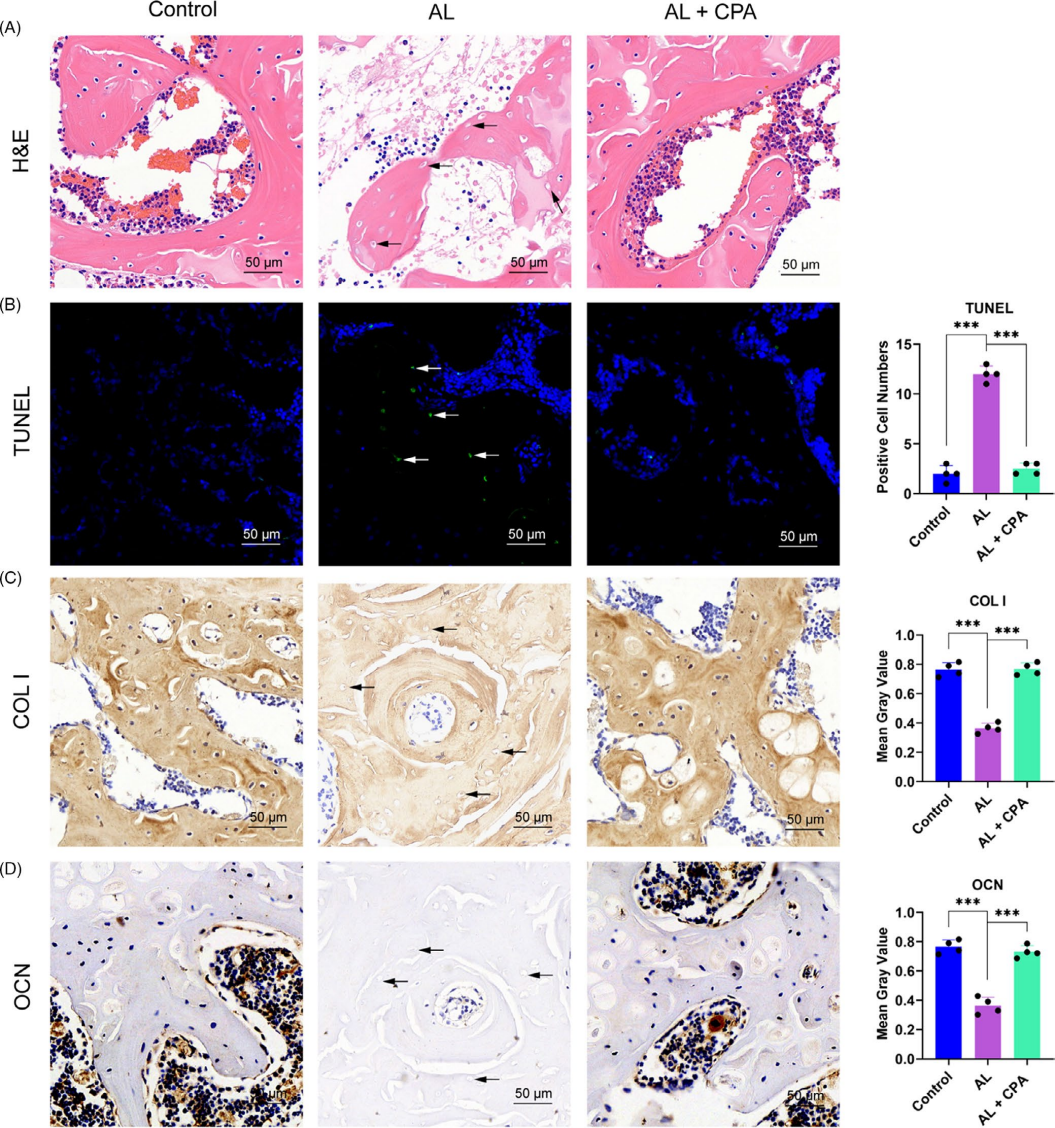
CPA alleviated alcohol‐induced ONFH in the rat model. (A) H&E staining indicated obvious osteonecrosis in the alcohol group. Empty lacunae in the subchondral trabeculae were marked by black arrows. (B) TUNEL indicated increased apoptosis in the alcohol group, which was alleviated by CPA treatment. The TUNEL‐positive cells were marked by white arrows. (C‐D) Immunohistochemical staining of COL I and OCN of the femoral head. The alcohol group had much weaker staining for COL I and OCN‐positive cells (black arrows) and extracellular matrix, while the staining was significantly improved with CPA administration

Osteonecrosis is characterized by the presence of pyknosis of nuclei in trabeculae accompanied with bone marrow cell necrosis or diffuse empty lacunae.^30^ The femoral head in the alcohol group displayed large pyknosis of nuclei in the trabeculae with bone marrow hematopoietic cellular debris in medullary spaces and diffused empty lacunae (Figure 6A). However, there were only marginal pathological changes in the alcohol group, but no apparent histopathological changes in the control group (Figure 6A). TUNEL staining was performed to detect apoptosis which is the most distinct manifestation of ONFH. The alcohol group had more positive staining cells with karyopyknosis and nuclear fragmentation, while the AL + CPA group had much fewer apoptotic cells (Figure 6B). Finally, immunohistochemical staining of COL I and OCN indicated that the alcohol group had obviously decreased osteogenic activity evidenced by significant positive staining of COL I and OCN, while alcohol‐induced inhibitory osteogenic activity was reversed by co‐treatment with CPA (Figure 6C and 6D).

**Figure 7:**
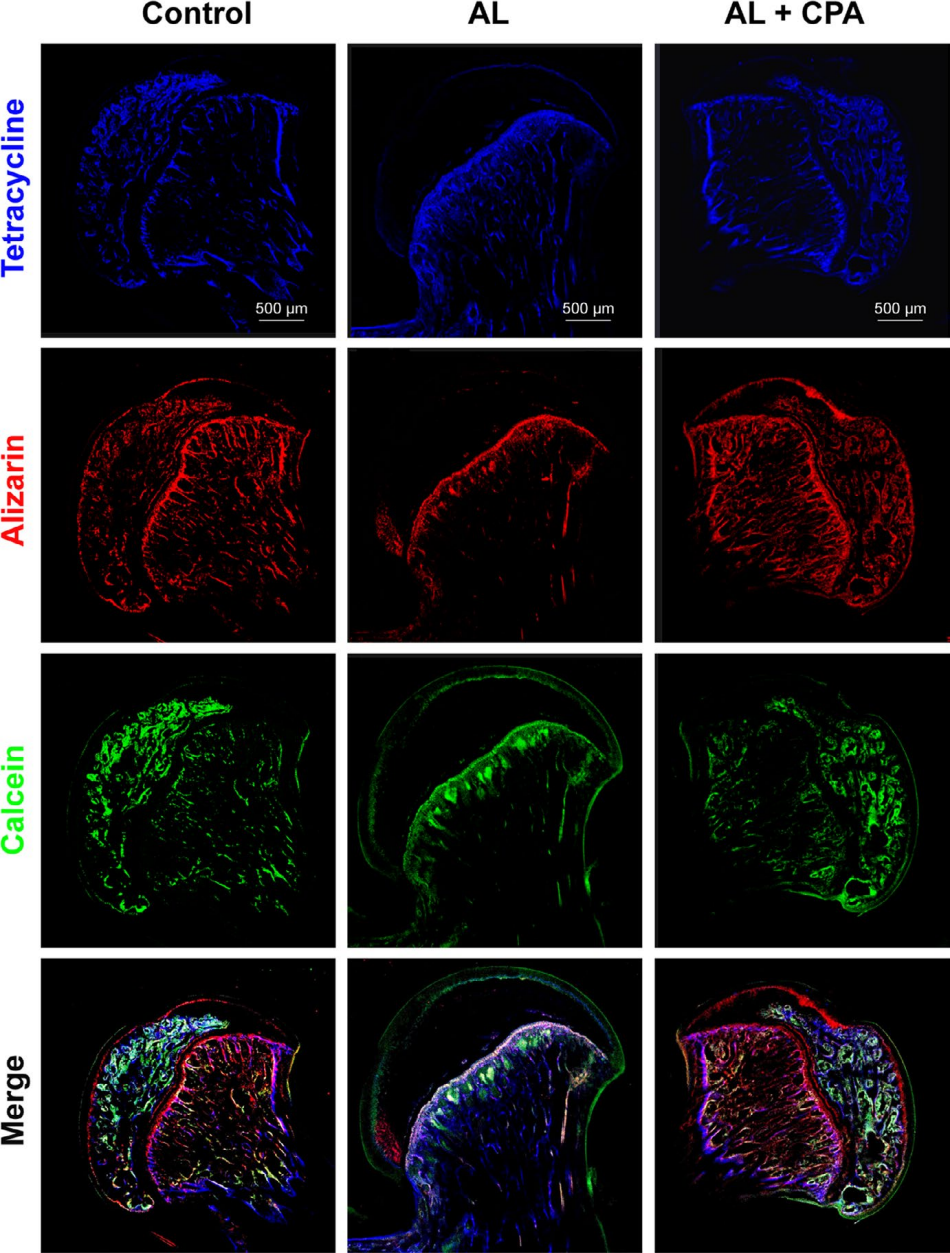
The protective effects of CPA against alcohol‐induced ONFH in the rat model. There was significantly decreased new bone formation in alcohol‐treated group revealed by the fluorochrome labelling, while the pharmacotherapy with CPA critically restored bone formation capacity

The dynamic bone formation and mineralization in the femoral head was monitored by fluorochrome labelling with tetracycline, Alizarin red S and calcein‐AM. The femoral head in the control group had a stronger fluorochrome labelling with tetracycline (blue), alizarin red S (red) and calcein (green) in a broader area of the subchondral trabeculae. However, the femoral head treated with alcohol had significantly decreased new bone formation and impaired bone homoeostasis as evidenced by much weaker fluorochrome labelling in the subchondral trabeculae (Figure 7). On the contrary, a much broader area of subchondral trabeculae was stained by fluorochrome labelling in the alcohol + CPA group, indicating the beneficial effect of CPA on the alcohol‐induced ONFH (Figure 7).

## 4 DISCUSSION

Piles of epidemiologic and multicentric studies have demonstrated that a large portion of ONFH patients have the history of alcohol overuse.^3^, ^31^, ^32^ Although there has been a lot of research intending to reveal the pathogenesis of ONFH, the exact mechanism of ONFH is still not fully understood. Previous studies indicated that osteogenesis capacity was remarkably impaired in ONFH, and both the number of BMSCs and the potential of osteogenic differentiation were obviously decreased.^30^, ^33^, ^34^, ^35^ In the present study, the results showed that the proliferation of BMSCs was inhibited by alcohol, and the osteogenic differentiation of BMSCs was dramatically retarded.

Abundant evidence has accumulated indicating that PI3K/AKT pathway is a central regulator in the osteogenesis of BMSCs.^36^, ^37^, ^38^, ^39^ Notably, PI3K/AKT pathway is involved in the osteogenic differentiation of alcohol‐induced bone loss in our previous work as well.^28^ Our results indicated that both levels of PI3K and phosphorylated AKT were remarkably decreased by alcohol. In this study, CPA could rescue the inhibition of alcohol on the proliferation and osteogenic differentiation of BMSCs. Previous studies indicated that CPA functioned in many cellular processes *via* PI3K/AKT signalling pathway.^25^, ^40^, ^41^, ^42^ Intriguingly, co‐treatment with CPA could significantly abolish the alcohol‐induced inhibition on PI3K and p‐AKT expression in BMSCs. The selective antagonist LY294002 further demonstrated that PI3K/AKT pathway critically contributed to the therapeutic role of CPA in the alcohol‐impaired osteogenic differentiation.

The pathomorphological changes of ONFH revealed extravascular fat deposition, characterized by enlarged cellular size and increased number of adipocytes.^35^, ^43^, ^44^ Therefore, several hypotheses have been proposed for the pathogenesis of ONFH, including fat emboli, disordered fat metabolism and abnormal intraosseous pressures due to intramedullary lipid accumulation.^43^ It is well known that BMSCs can differentiate into osteoblast, chondrocyte and adipocyte.^45^ Prior studies indicated that only a few of BMSCs can differentiate into adipocytes in normal culture condition.^46^ In our study, BMSCs inclined to the differentiation of massive adipocytes while the induction to osteoblasts was critically inhibited when treated with alcohol. A myriad of triglyceride‐containing vesicles could aggregate in the BMSCs‐differentiated adipocytes. The increased volume of lipid vacuoles might reduce arterial perfusion, with concomitant increase in intraosseous pressure, venous stasis and hypertension, which may contribute to the foundation of the alcohol‐induced ONFH.^10^, ^46^ Moreover, the direct toxic effect of ethanol and its metabolites cause a fatty liver and other alcoholic liver injury, resulting in impaired hepatocyte function and deteriorated fatty acid oxidation.^47^ Alcohol‐induced metabolic disorders may lead to massive fatty vesicles in the circulation and impair the fragile blood supply of the femoral head.^43^ Fortunately, CPA could substantially retard the promotion of adipogenic differentiation of BMSCs irritated by alcohol; therefore, CPA might protect the alcohol‐induced ONFH *via* modulating the alcohol‐induced adipogenesis of BMSCs. AMPK pathway is a “cellular fuel gage” which functions in rapid changes for the mastery of fatty acid metabolism.^20^ The existing research indicated that alcohol could affect cholesterol and lipid homoeostasis *via* the regulation of AMPK pathway,^48^ which is also the main pharmacological mechanism of CPA.^20^ We found that alcohol significantly decreased the level of p‐AMPK, while CPA reversed it. Further evidence from selective antagonists, DMP and LY294002, indicated that AMPK and PI3K/AKT pathway greatly contributed to the differentiation direction of BMSCs under the alcohol stress. CPA could alleviate the pathological changes of ONFH *via* augmenting osteogenesis and retarding adipogenesis in the scenario of ethanol administration.

in vivo, animals in the alcohol group exerted typical pathological changes of ONFH as evidenced by micro‐CT scanning and HE staining, while only mild osteonecrosis was observed in the rats with the administration of CPA. Additionally, there were more apoptotic cells in the ethanol group, indicating that apoptosis is an imperative change in the development of ONFH. However, CPA played a protective role in alleviating cell death and preventing alcohol‐induced ONFH. Moreover, previous work has indicated that CPA is a quite safe drug, even the high dose (30 μg/mL) of which has no significant disturbance in chromosomal aberrations,^49^ and the accumulation of CPA metabolites is quite low in the liver and kidney.^50^ Our results also demonstrated that CPA was well tolerated as evidenced by no changes in daily behaviour, survival rate and body weight in rats.

In summary, CPA is convincingly demonstrated to be beneficial to the alcohol‐induced ONFH, largely due to its capacity of shifting the differentiation tendency of BMSCs, and to ameliorate radiological and histological features of the femoral head in rats. Hence, CPA may become a potential pharmacotherapeutic herb extract for the prevention of the alcohol‐induced ONFH.

## CONFLICT OF INTEREST

The authors declare no conflicts of interests.

## AUTHOR CONTRIBUTIONS

Y. Chen, C. Zhang and Y. Gao designed research protocol. H. Yu, P. Liu, D. Zhu, J. Yin, Q. Yang and Y. Huang conducted research. H. Yu, P. Liu, D. Zhu, Q. Yang, Y. Chen and Y. Gao analysed data. H. Yu, Y. Chen and Y. Gao wrote the manuscript. C. Zhang and Y. Gao critically revised the manuscript. All authors approved the final manuscript.

## Data Availability

All data that support the findings of this study are available from the corresponding author upon request.
